# Five-Year Follow-Up of Distal Tibia Bone and Foot and Ankle Trauma Treated with a 3D-Printed Titanium Cage

**DOI:** 10.1155/2019/7571013

**Published:** 2019-11-26

**Authors:** Eugene C. Nwankwo, Fangyu Chen, Dana L. Nettles, Samuel B. Adams

**Affiliations:** Department of Orthopaedic Surgery, Duke University Medical Center, USA

## Abstract

Large bone defects from trauma or cancer are difficult to treat. Current treatment options include the use of external fixation with bone transport, bone grafting, or amputation. These modes of therapy continue to pose challenges as they are associated with high cost, failure, and complication rates. In this study, we report a successful case of bone defect treatment using personalized 3D-printed implant. This is the longest known follow-up using a 3D-printed custom implant for this specific application. Ultimately, this report adds to existing literature as it demonstrates successful and maintained incorporation of bone into the titanium implant. The use of patient-specific 3D-printed implants adds to the available arsenal to treat complex pathologies of the foot and ankle. Moreover, the technology's flexibility and ease of customization makes it conducive to tailor to specific patient needs.

## 1. Introduction

Despite advances in modern orthopaedic traumatology, bone loss following high-energy trauma remains a challenge to treat. Traditional treatment modalities include bone transport using an external fixator [1], cancellous bone grafts with and without tissue transfer [2], transfer of vascularized fibular osteomyocutaneous flaps [3], and the Masquelet technique and other two-stage reconstructions [4].

3D printing, also called additive manufacturing, is the process of creating digitally designed objects by deposition of materials in sequential layers [5]. This rapidly advancing field has created access to almost limitless 3D structures. These structures can be manufactured from a variety of materials, such as plastics, metals, and living cells [5]. The ability to customize objects for 3D printing for personalized medical applications is based on the ability to design implants from a patient's own anatomy (taken from CT and/or MRI imaging). This makes additive manufacturing an attractive alternative to treat critical-sized bone defects of the foot and ankle.

Previously, we reported early follow-up on a case of limb salvage using a 3D-printed patient-specific titanium cage in a 46-year-old female who sustained traumatic distal tibia segmental bone loss and multiple additional foot fractures [6]. Since the use of this technology is still relatively new with unknown efficacy, it is important to report successes and failures as well as longer term follow-up. Here, we report a five-year follow-up with imaging and patient-reported outcomes. This is the longest known follow-up using a 3D-printed custom implant for this application and adds to the literature by demonstrating successful and maintained bone incorporation into the implant.

## 2. Case Report

**Figure 1:**
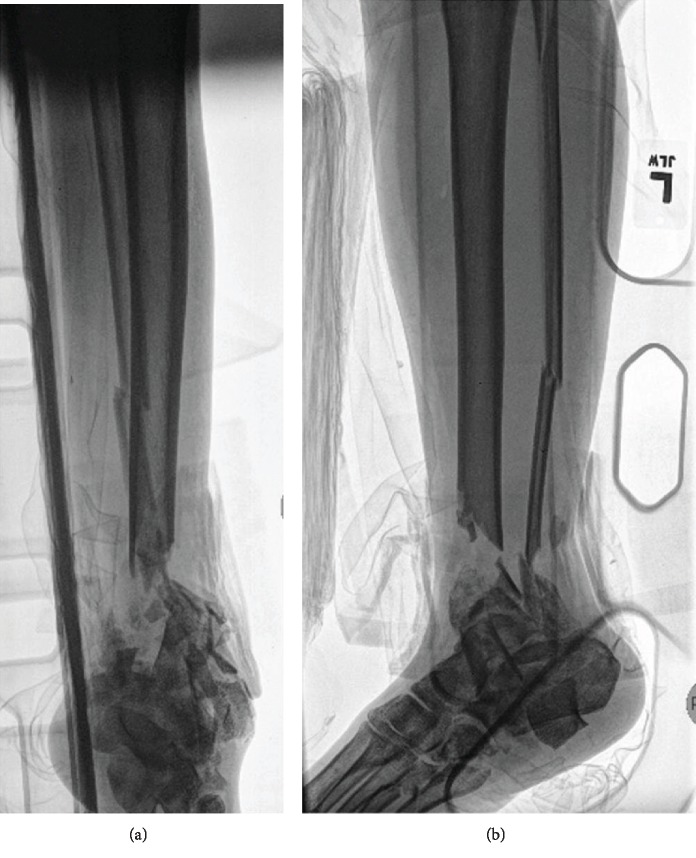
Anteroposterior (a) and lateral (b) radiographs taken at the time of the original injury demonstrating distal tibia bone loss and multiple foot fractures.

The 46-year-old female was the driver of a car that rolled over. She sustained an open distal tibia fracture with substantial distal-third tibia bone loss (Figure 1). Other bony injuries included comminuted fractures of the fibula and talar body. Moreover, bony injuries to the foot included fractures of the posterior facet of the calcaneus, second through fifth metatarsal fractures, and cuboid. She was neurovascularly intact distal to the segmental bone loss. The initial surgery included irrigation and debridement, placement of an external fixator and an antibiotic-impregnated polymethylmethacrylate spacer.

Amputation and multiple limb salvage options were discussed with the patient and primary surgeon (SBA) via the shared decision-making process. The patient opted to proceed with limb salvage via arthrodesis of the tibia to the hindfoot using a custom 3D-printed titanium cage (4WEB Medical, Frisco, TX, USA) and an intramedullary rod. The patient was non-weight bearing for six weeks after surgery. This period was followed by a six-week period of limited weight bearing in a cast. She was then transitioned to full weight bearing in a boot brace over the last six weeks.

**Figure 2:**
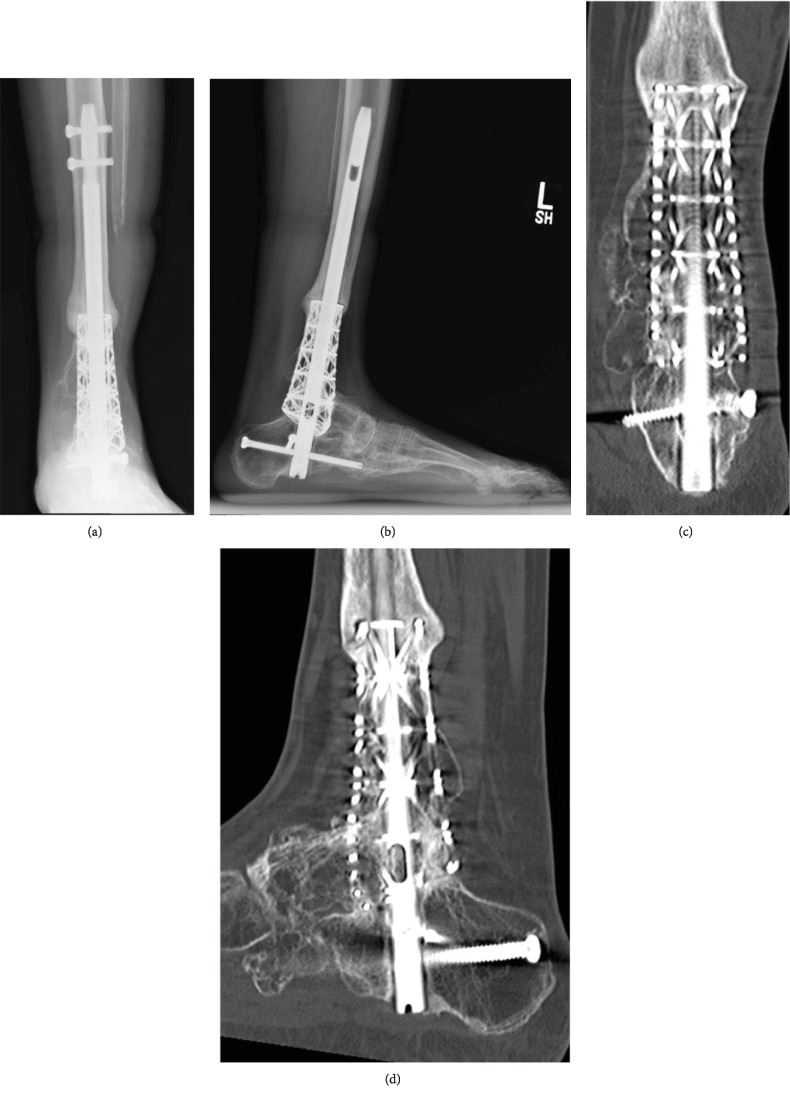
Anteroposterior (a) and lateral (b) radiographs of the lower leg and hindfoot 48 months after definitive surgery. Coronal (c) and sagittal (d) CT scan images from the same timepoint demonstrating bone growth into the cage from the tibia, talus, and calcaneus.

**Figure 3:**
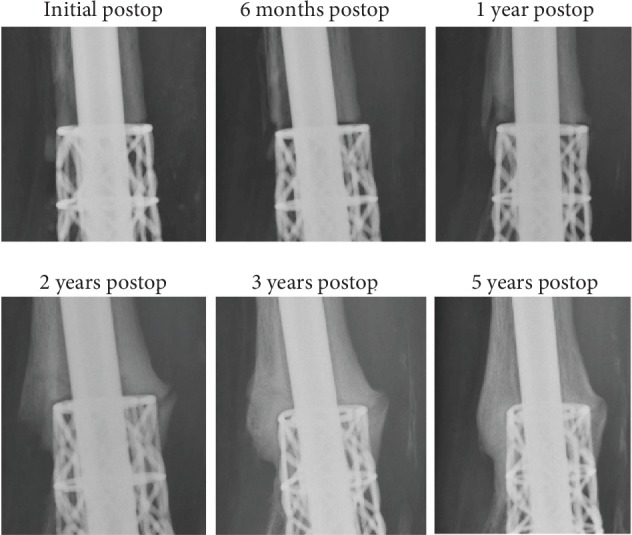
Serial radiographs demonstrating increased bone deposition at the interface of the distal tibia diaphysis and the proximal extent of the cage.

She was followed closely with routine plain radiographs approximately every 6 to 12 weeks and CT scans every 6 months. By six months after surgery, the patient had returned to special needs elementary school teaching without any ambulatory aids and with regular shoe wear as tolerated. Her only pain was transient heel pad pain from the intramedullary rod insertion site. The patient's most recent follow-up was at 60 months after surgery. Plain radiographs and CT scan (Figure 2) demonstrated successful bone incorporation of the talus, calcaneus, and tibia. Proof of progressive bone growth incorporation of the cage can be seen with serial radiographs of the proximal interface of the cage and native tibia (Figure 3). Preoperatively, as well as, at the recent follow-up, the Foot and Ankle Ability Measure (FAAM) and AOFAS ankle-hindfoot score were administered. Because she initially presented after trauma and in an external fixator (when the preoperative outcome measures were given), only her most recent follow-up scores are presented here.

The FAAM has two subscales and asks patients to rate their level of functionality. Her responses were as follows. Her FAAM activities of daily living subscale score was 79 (66 out of 84 total points), and she rated her functionality during activities of daily living as 85% of her baseline function prior to her injury. Her FAAM sports subscale score was 46 (13 out of 28 total points), and she also rated her current level of function during sports-related activities as 85% of what it was prior to her injury. She also responded with a rating of “nearly normal” regarding her current level of function.

Her AOFAS ankle-hindfoot scale score was 71 out of 100. She reported “mild, occasional” pain. Most of the reduction in this score came from the loss of sagittal and hindfoot motion.

## 3. Discussion

Treating segmental bone loss of the lower extremity has been a major challenge for orthopaedic surgeons. Reconstruction methods with autografts or allografts have had variable success [1–3, 7, 8]. Autografts have the disadvantage of donor site morbidity and limited shape and quantity, especially when dealing with large bone defects [9, 10]. On the other hand, allografts are also potentially limited in their shape and size. Allografts are also less osteogenic with higher rates of nonunion with the theoretical risk of disease transmission [11, 12]. Moreover, both are known to undergo late collapse leading to structural failure and can be limited by the ability to truly achieve the correct shape for reconstruction based on limitations in human osseous anatomy from which the graft is obtained [11, 13]. Additive manufacturing could solve many of the problems posed by both autograft and allograft.

This five-year follow-up report describes successful limb salvage with the use of a FDA-approved and custom-designed 3D-printed titanium implant following segmental bone loss of the foot and ankle. The present decade has seen rapid advancement in 3D printing technologies and increases in applications for medicine, contributing to the growth in available patient-specific and customizable avenues of care. Current applications include patient-specific instrumentation, 3D models for surgery planning, navigational templates, total knee implants, and customizable talar implants [14–21]. 3D printing technology ushers in a new era in the treatment of patients previously relegated to complex limb salvage or amputation.

Literature has demonstrated that the cost of amputations over a lifetime serves as more of a deterrent than previously thought. This discrepancy in cost can be attributed to the use of prosthetics [22]. Costs of limb salvage in the form of Ilizarov bone transport are less but still considerable [23]. Though still expensive as a new technology, the future holds great promise for 3D printing in the value proposition [24]. Emerging advances that have the ability to directly impact surgical applications include technologies with the ability to bring scanning and printing capabilities into the OR [25], handheld printing “pens” for smaller defects [26, 27], arthroscopic printing of cells [28], and direct thermal printing to defects [29].

3D printing has several disadvantages as well that may preclude custom implants from widespread adoption. First, 3D-printed implants are expensive. However, these technologies have the ability to significantly decrease turnaround times, thus decreasing the need for large inventory stocking and the associated space used to house the unused implants, driving down overhead and healthcare costs. As the technology is more broadly accepted with multiple device companies adopting its usage, its costs will subsequently decrease. Another disadvantage is unknown capacity for bone ingrowth and stress shielding. However, one of the main purposes of this case report was to detail the ability for bone ingrowth and the lack of stress shielding that occurred in this patient (Figure 3). As with other metal implants, infection and biofilm formation is a major concern. Currently, 3D-printed implants are not immune from contamination and should not be used in infected patients.

As additive manufacturing technology continues to expand and spreads more widely and as technical experiences are shared, reports of successful use of 3D-printed implants in surgical care are increasing. Hsu and Ellington first reported the use of a 3D-printed titanium cage truss for use in a persistent tibial nonunion with excellent results at one year [30]. Likewise, So and coworkers recently reported on the use of this same technology for application in three different foot or ankle procedures and also report excellent outcomes out to 17 months [31]. We recently published the largest known follow-up study of this kind that included a cohort of fifteen consecutive patients treated with custom-designed 3D-printed implant cages for severe bone loss, deformity correction, and/or arthrodesis procedures at a single institution and performed by a single surgeon. A minimum of 1-year clinical and radiographic follow-up was required, with a mean follow-up of 22 months (range, 12-48 months). There was a significant improvement in pain and all functional outcome score measures. All patients who went on to fusion were satisfied with their surgery. There were two failures, one infection and one nonunion, with an overall clinical success rate of 87% [32].

Several aspects of this case portended a successful outcome. First, despite severe trauma, the limb was sensate and well perfused. Second, there was no infection. The authors do not encourage the use of this kind of reconstruction in limbs with neurovascular compromise or in the setting of active infection or a history of infection. Finally, the patient was well informed about the potential risks and complications of the procedure and maintained a high compliance with the postoperative protocol.

## 4. Summary/Conclusion

Here, we reported the longest known follow-up of successful implantation and bony incorporation of a 3D-printed patient-specific titanium cage. Large lower extremity bony defects, complex foot and ankle deformities, and high-risk arthrodesis situations can be difficult to treat. These challenging pathologies often require a critical-sized and/or shaped structural bone void filler which may not be available with autograft or allograft bone. The use of patient-specific 3D-printed titanium implants offers a new technology that can address a variety of bone defects and deformities to successfully treat a variety of difficult to treat pathologies in foot and ankle and lower extremity surgery. Furthermore, the flexibility and ease of customization of implants allow for patient-specific needs to be met and planned for preoperatively. The case presented herein is the first published observation of long-term success for the treatment of large bone defect using a 3D-printed implant. Thus, the techniques used in this case were not based on prior established evidence. The surgical interventions utilized here are not mainstream and, as such, should be used with caution when considered in the treatment of other patients.
